# Case report: Rare heterozygous variant in the *NR5A1* gene causing 46,XY complete gonadal dysgenesis with a non-communicating rudimentary uterus

**DOI:** 10.3389/fmed.2024.1441990

**Published:** 2024-08-01

**Authors:** Toru Sasaki, Shinji Suzuki, Masanori Ono, Akiko Yamamoto, Masato Bingo, Gaku Yamanaka, Masahiko Kuroda, Natsuko Inagaki, Hirotaka Nishi

**Affiliations:** ^1^Department of Obstetrics and Gynecology, Tokyo Medical University, Tokyo, Japan; ^2^Department of Pediatrics, Tokyo Medical University, Tokyo, Japan; ^3^Department of Clinical Genetics Center, Tokyo Medical University, Tokyo, Japan; ^4^Department of Laboratory Medicine, Tokyo Medical University, Tokyo, Japan; ^5^Department of Molecular Pathology, Tokyo Medical University, Tokyo, Japan; ^6^Department of Cardiology, Tokyo Medical University, Tokyo, Japan

**Keywords:** case report, complete gonadal dysgenesis, disorder of sex development, genomic structural variants, pathogenicity

## Abstract

The nuclear receptor subfamily 5 group A member 1 (*NR5A1*) gene encodes NR5A1, also known as steroidogenic factor 1, a crucial transcriptional factor regulating adrenal and gonadal development and function. Although pathogenic variants in *NR5A1* are known to cause a spectrum of disorders of sex development (DSD), individuals with 46,XY DSD with fully female internal and external genitalia are relatively rare. Herein, we present the case of a patient with 46,XY complete gonadal dysgenesis (CGD) who had a non-communicating rudimentary uterus due to a c.132_134del (p.Asn44del) heterozygous in-frame-deletion in *NR5A1* that was diagnosed while treating a pelvic mass in which gynecological malignancy could not be disregarded. Unlike two previous cases with the p.Asn44del variant, this case presented with CGD, a severe DSD phenotype, and we found that the oligogenic inheritance of DSD-causative genes such as *SRY*, *DHX37*, *SLC26A8*, and *CFTR* may have affected the severity of the clinical phenotype.

## 1 Introduction

Disorders of sex development (DSD) are defined as congenital conditions in which the development of chromosomal, gonadal, and anatomic sex is atypical (1–3). One such DSD is 46,XY DSD, which has an incidence rate of approximately 1 in 20,000 births (3, 4). Based on the contents of sex chromosomes, DSD is classified into the following three categories: sex-chromosome DSD, 46,XX DSD, and 46,XY DSD (5, 6). 46,XY complete gonadal dysgenesis (CGD), also known as Swyer syndrome, is a subtype of 46,XY DSD that results from variants in pivotal testis-determining genes in a phenotypic female with internal Müllerian structures and bilateral streak gonads (7, 8). One such gene is the nuclear receptor subfamily 5 group A member 1 (*NR5A1*) gene that encodes NR5A1, also known as adrenal-4 binding protein or steroidogenic factor 1 (SF-1), a crucial transcriptional factor regulating proper adrenal and gonadal development and function (9–12). Although *NR5A1* variants can lead to a range of DSD phenotypes, individuals with fully female internal and external genitalia (46,XY CGD) are not common (9, 11).

In this study, we describe the rare case of a patient with 46,XY CGD caused by a heterozygous c.132_134del (p.Asn44del) variant in the *NR5A1* gene, which was discovered during treatment for a pelvic mass with possible gynecological malignancy.

## 2 Case description

### 2.1 Clinical history

This study was approved by the Ethics Committee of the Tokyo Medical University Hospital (Tokyo, Japan). Written informed consent was obtained from the patient. The proband in this study was the first child of non-consanguineous, healthy parents with no family history of DSD or adrenal dysfunction. The patient was born at term without genital ambiguity, with normal weight and length. While there were no marked abnormalities in the patient’s primary and secondary sexual characteristics, the patient had visited a hospital in Korea for primary amenorrhea at the age of 15, where DSD was suspected and a chromosome test was performed. Consequently, the patient was diagnosed with 46,XY DSD with typical female genitalia.

At the initial visit, laboratory analysis indicated high luteinizing and follicle-stimulating hormone levels, slightly high testosterone levels, high dehydroepiandrosterone sulfate levels, normal sex hormone-binding globulin levels, low estradiol levels, and anti-Müllerian hormone (AMH) levels below detection sensitivity. In addition, pelvic magnetic resonance imaging (MRI) revealed a vagina, a hypoplastic uterus with an undeniable rudimentary uterus, and no evidence of bilateral adnexa. Ten months after the initial visit, the patient underwent a laparoscopic bilateral gonadectomy to reduce the risk of malignancy arising from the gonads. The pathological diagnoses of the right and left gonads were atrophic testicular and salpingeal tissues, respectively. Postoperatively, estrogen replacement therapy was administered, followed by hormone replacement therapy with a combination of estrogen and progestin.

**Figure 1:**
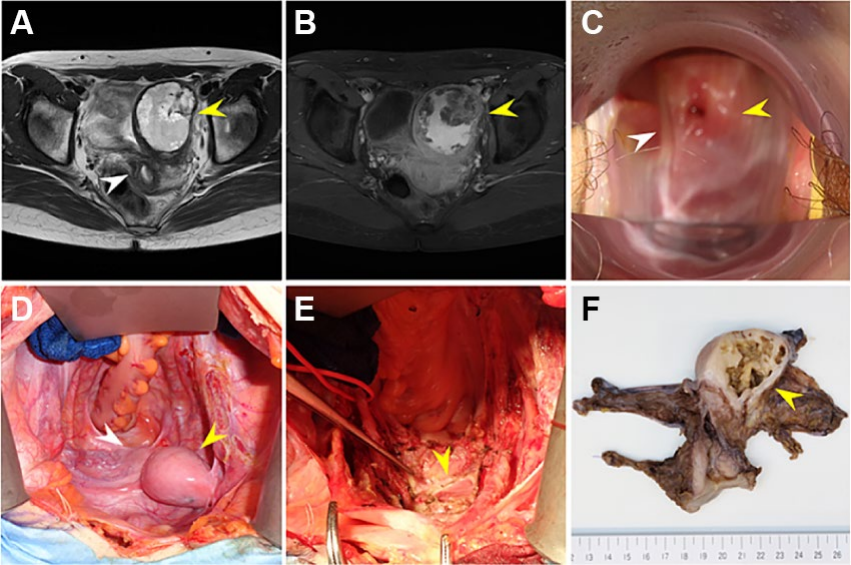
**(A)** T2-weighted pelvic magnetic resonance imaging (MRI) showing a vagina, a hypoplastic uterus (white arrowhead), and a 10-cm left-pelvic cystic lesion with partially solid components (yellow arrowhead). **(B)** Fat-suppression T1-weighted pelvic enhanced MRI with contrast effects showing that the left-pelvic cystic lesion (yellow arrowhead) has hematogenous contents and partially solid components, suggesting that the possibility of malignancy could not be disregarded. **(C)** Vaginal speculum examination upon surgery revealing an incompetent vaginal septum (white arrowhead) and an atrophic single uterine cervix (yellow arrowhead). **(D)** Laparotomy revealing the following intraoperative findings: the left-pelvic tumor (yellow arrowhead) is contiguous with the atrophic uterus (white arrowhead) and there are no ascites or obvious disseminated lesion. **(E)** Intraoperative findings after removing the uterus and left-pelvic tumor: the left-pelvic tumor was removed as one piece of tissue with the atrophic uterus, without perforation (the yellow arrowhead shows a single vagina). **(F)** Macroscopic findings of the surgical specimen: the permanent pathologic diagnosis indicates that the pelvic mass was a non-communicating rudimentary uterus with a retained uterine hematoma, with no malignant findings.

At the age of 23, the patient moved to Japan and continued hormone replacement therapy at a gynecologic clinic. Although no lower abdominal pain or dysmenorrhea were observed during treatment, transvaginal ultrasonography performed for screening revealed a 10-cm cystic mass with undeniable gynecologic malignancy on the left side of the pelvis, after which the patient was referred to our institution for further examination and treatment. Pelvic enhanced MRI with contrast effects showed a vagina, a hypoplastic uterus, and a 10-cm left-pelvic cystic lesion with hematogenous contents and partially solid components (Figures 1A,B). In addition, a vaginal speculum examination performed at the first visit to our institution revealed an incompetent vaginal septum and an atrophic single cervix (Figure 1C). Based on these findings, a malignant tumor arising from the gonadal remnant tissue or endometrial cancer in the rudimentary uterus was diagnosed. As the patient did not wish to preserve her fertility, informed consent was obtained and a laparotomy was performed to facilitate a definitive diagnosis and treatment course.

### 2.2 Intraoperative findings

Intraoperative findings revealed that the left-pelvic tumor was contiguous with the atrophic uterus, with no marked disseminated lesion. Consequently, one piece of tissue containing both the tumor and atrophic uterus was removed without perforation (Figures 1D,E). Intraoperative rapid pathologic diagnosis indicated that the pelvic mass was a non-communicating rudimentary uterus with a retained uterine hematoma and no malignant findings. Postoperatively, the permanent pathologic diagnosis was confirmed to be similar to the intraoperative diagnosis (Figure 1F); therefore, estrogen replacement therapy was resumed.

### 2.3 DNA extraction

Genomic DNA was extracted from peripheral blood mononuclear cells using the DNeasy Blood & Tissue Kit (Qiagen, Hilden, Germany) according to the manufacturer’s instructions. The samples were mixed with 200 μL of AL buffer and 200 μL of ethanol (96–100%), after which they were incubated at 56°C for 10 min. The samples were then mixed with 200 μL of ethanol and the supernatants were subjected to a washing process using a spin column, according to the manufacturer’s instructions. DNA quality and quantity were verified using Qubit 2.0 fluorometry (Life Technologies, Carlsbad, California, USA) and the Agilent 2000 TapeStation.

### 2.4 Sequencing and analysis

Exome enrichment of genomic DNA was performed using the SureSelect Human All Exon V7 Kit (Agilent, Santa Clara, California, USA), and the Illumina NextSeq 2000 (San Diego, California, USA) platform was used for massive parallel sequencing. In addition, data analysis was performed using the DRAGEN Germline application version 3.8.4 (Illumina, San Diego, USA), with all parameter settings set to default, and ANNOVAR was used for the annotation.

*In silico* analysis was performed using prediction tools from version 4.2 of the dbNSFP database,^1^ and the SIFT, Polyphen-2, and combined annotation-dependent depletion scores were used to interpret the variant pathogenicity. The AlphaMissense score was also used for prediction. The following criteria were used for diagnostic filtering: (1) retain variants on the list of 83 known DSD-causative genes from the literature (13), (2) retain variants with allele frequencies <1% in the Genome Aggregation Database,^2^ (3) exclude synonymous variants, and (4) retain variants classified as “Likely Pathogenic” or “Pathogenic” according to the American College of Medical Genetics and Genomics classification criteria. After filtering, the remaining candidates were validated using conventional Sanger sequencing (9–12).

All protocols used during sequencing and analysis were performed according to the manufacturer’s instructions. Genetic testing was performed after obtaining written informed consent, and a clinical geneticist provided genetic counseling.

### 2.5 Genetic findings

**Figure 2:**
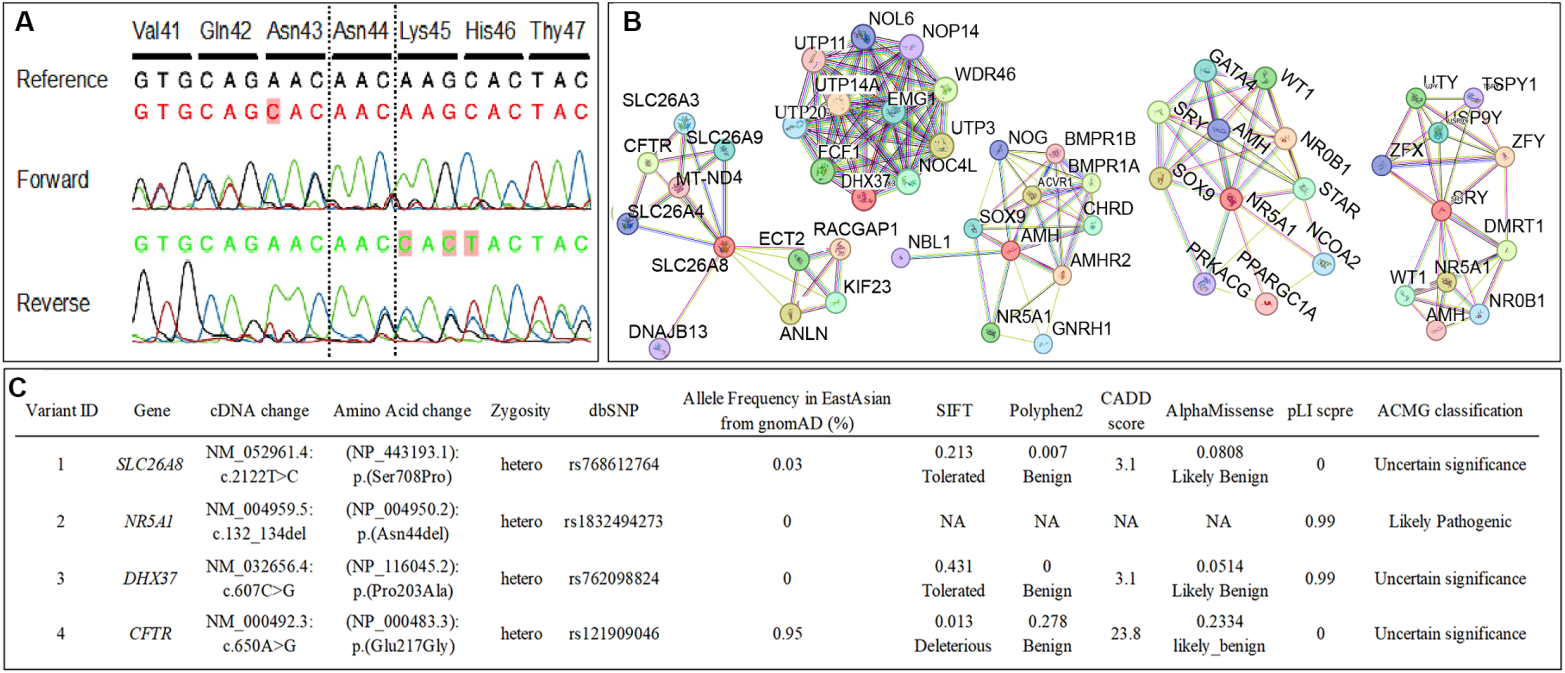
**(A)** The conventional Sanger sequencing for *NR5A1* in this case confirms a c.132_134del (p.Asn44del) heterozygous in-frame-deletion in *NR5A1*. The bases that differed from the reference sequence are colored in red. **(B)** The functional protein association networks for *NR5A1*, *SRY*, *AMH*, *DHX37*, and *SLC26A8* determined using STRING. **(C)** The nonsynonymous variants in previously reported disorders of sex development (DSD)-causative genes and candidate genes of functional protein association networks for *NR5A1*, *SRY*, *AMH*, *DHX37*, and *SLC26A8* determined using whole-exome sequencing. The American College of Medical Genetics and Genomics criteria classified the *NR5A1* variant as pathogenic/likely pathogenic, whereas the variants in *SRY*, *DHX37*, *SLC26A8*, and *CFTR* were classified as benign/likely benign.

As the patient wished to search for the etiologically responsible genes and evaluate the risk of developing other concomitant diseases, whole-exome sequencing (WES) was performed. Notably, WES detected some variants of previously known DSD-causing genes, including the c.132_134del (p.Asn44del) heterozygous in-frame-deletion in *NR5A1* (Figure 2A) and heterozygous nonsynonymous variants of unknown significance (VUS) in *DHX37* and *SLC26A8*, which may be involved in DSD development. The c.465C > T (p.Ser155=) homozygous synonymous variant in *SRY* was manually retrieved as a candidate phenotype-related variant since *SRY* is a crucial DSD-causative gene that is also closely related to *NR5A1* (11, 14, 15). *In silico* analysis indicated that the variant in *NR5A1* was pathogenic or likely pathogenic, whereas the variants in *SRY*, *DHX37*, and *SLC26A8* were benign or likely benign (Figure 2C). Furthermore, by investigating protein–protein interactions for *NR5A1*, *SRY*, *AMH*, *DHX37*, and *SLC26A8* using the STRING database,^3^ we detected an additional rare variant in *CFTR* that encodes DSD-causative proteins in these associated networks (Figures 2B,C).

## 3 Discussion

NR5A1/SF-1 is expressed in the bipotential gonad and modulates genes such as *SRY* and *SOX9* to regulate and maintain male determination (16, 17). To date, various *NR5A1* variants related to 46,XY DSD have been reported, and a wide range of phenotypes have been observed (13, 18). Some cases of *NR5A1* variants have normal phenotypes, while other DSD-associated gene variants are often comorbid when combined with *NR5A1* variants in patients with DSD with a severe phenotype (15, 19). In the present case, variants in *NR5A1*, *SRY*, *DHX37*, and *SLC26A8*, which could be related to the pathogenesis of DSD, were identified using WES. However, *in silico* analysis revealed that the variants in genes other than *NR5A1* were not pathogenic.

There are two previous reports of c.132_134del (p.Asn44del) heterozygous in-frame deletions in *NR5A1*—the etiology in this case—in patients with 46,XY DSD. Both previous cases did not have any comorbid variants of other DSD-causative genes and were phenotypically male with no Müllerian component, which was different from our case in which the presence of Müllerian structures suggested a defect in fetal Sertoli cell differentiation and AMH secretion (13, 14). Notably, a previous study revealed that haploinsufficiency cannot sufficiently explain broad clinical phenotypes because patients with the same genetic variants may present with different clinical features, even within the same family linage (15). The same genetic changes in *NR5A1* can display a variable expression of clinical phenotypes (20). One explanation for this may be oligogenic inheritance, in which multiple individually non-deleterious hits may contribute to a DSD phenotype (15, 21, 22). In the present case, our *in silico* analysis results indicated that the identified variants in *SRY*, *DHX37*, *SLC26A8*, and *CFTR* coexisting with the variant in *NR5A1* were not likely to be involved in the severe phenotype. However, given that the oligogenic inheritance of DSD-causative genes might affect clinical phenotypes and that the two previous cases of p.Asn44del did not have as severe phenotypes as that in our case, it is difficult to completely disregard the involvement of these benign and VUS variants of DSD-causative genes. Notably, a previous study found a double variant in *NR5A1* and *DHX37* in 16% of a cohort of 25 individuals with 46,XY DSD, showing the potential contribution of these variants to female-type sex differentiation (4).

*CFTR* is the causative gene for both cystic fibrosis and the congenital bilateral absence of the vas deferens (23). A previous study on zebrafish has shown that *CFTR* is crucial in regulating primordial germ cell migration during early embryogenesis. Furthermore, ion exchange through the interaction between *CFTR* and *SLC26A8* is also involved in sperm fertilization (24), and these two ion exchange genes are expressed in human germ and Sertoli cells, which may modulate gonadal development (25); however, their relationship with the *SOX9* pathway is uncertain. We speculate that the total effect of these four variants on gonadal differentiation results in impaired fetal Sertoli cell differentiation and AMH secretion, which in turn results in a female phenotype different from that previously reported in cases with variants in *NR5A1*.

Although our results suggest that oligogenic inheritance may be involved in the phenotypic expression of DSD, the diagnostic yield of 46,XY DSD is currently approximately 24–46% (13, 18). Thus, focusing on known DSD-causative genes only might miss some other novel candidate genes. Another limitation of this study was that the analysis was based on WES and not whole-genome sequencing. Therefore, further investigation regarding the introns, promoters, enhancers, and epigenomes related to abnormal *NR5A1* expression levels and how these are associated with the broad spectrum of DSD phenotypes is required.

In conclusion, although *NR5A1* has a large effect on *SOX9* regulation, our case suggests that the phenotypic difference in patients with DSD is due to the sum of the effect sizes of other genes involved in gonadal differentiation. Therefore, an interpretive model incorporating differences in the effect sizes of gene groups associated with male differentiation is expected to be useful for future DSD genetic testing.

## Data availability statement

The datasets presented in this article are not readily available because of patient confidentiality. Requests to access the datasets should be directed to masanori@tokyo-med.ac.jp.

## Ethics statement

The studies involving humans were approved by Ethics Committee of the Tokyo Medical University Hospital. The studies were conducted in accordance with the local legislation and institutional requirements. The participants provided their written informed consent to participate in this study. Written informed consent was obtained from the individual(s) for the publication of any potentially identifiable images or data included in this article.

## Author contributions

TS: Formal analysis, Investigation, Writing – original draft, Writing – review & editing. SS: Formal analysis, Writing – original draft, Writing – review & editing. MO: Investigation, Project administration, Writing – original draft, Writing – review & editing. AY: Investigation, Writing – original draft, Writing – review & editing. MB: Investigation, Writing – original draft, Writing – review & editing. GY: Investigation, Writing – original draft, Writing – review & editing. MK: Investigation, Writing – original draft, Writing – review & editing. NI: Investigation, Writing – original draft, Writing – review & editing. HN: Investigation, Writing – original draft, Writing – review & editing.

## Footnotes

^1^ http://database.liulab.science/dbNSFP

^2^ https://gnomad.broadinstitute.org/

^3^ https://string-db.org/

## References

[1] Houk CP Lee PA. Update on disorders of sex development. Curr Opin Endocrinol Diabetes Obes. (2012) 19:28–32. doi: 10.1097/MED.0b013e32834edacb 22157406

[2] Fisher AD Ristori J Fanni E Castellini G Forti G Maggi M. Gender identity, gender assignment and reassignment in individuals with disorders of sex development: a major of dilemma. J Endocrinol Investig. (2016) 39:1207–24. doi: 10.1007/s40618-016-0482-0, PMID: 27287420

[3] Garcia-Acero M Moreno O Suarez F Rojas A. Disorders of sexual development: current status and Progress in the diagnostic approach. Curr Urol. (2020) 13:169–78. doi: 10.1159/000499274, PMID: 31998049 PMC6976999

[4] de Oliveira FR Mazzola TN de Mello MP Francese-Santos AP Lemos-Marini SHV Maciel-Guerra AT. DHX37 and NR5A1 variants identified in patients with 46,XY partial gonadal dysgenesis. Life. (2023) 13:1093. doi: 10.3390/life13051093 37240737 PMC10222664

[5] Wherrett DK. Approach to the infant with a suspected disorder of sex development. Pediatr Clin N Am. (2015) 62:983–99. doi: 10.1016/j.pcl.2015.04.011, PMID: 26210628

[6] Hughes IA Houk C Ahmed SF Lee PA L.C. Group E.C. Group. Consensus statement on management of intersex disorders. Arch Dis Child. (2006) 91:554–63. doi: 10.1136/adc.2006.098319, PMID: 16624884 PMC2082839

[7] Ostrer H. Sexual differentiation. Semin Reprod Med. (2000) 18:041–50. doi: 10.1055/s-2000-13474 11299518

[8] Michala L Goswami D Creighton SM Conway GS. Swyer syndrome: presentation and outcomes. BJOG. (2008) 115:737–41. doi: 10.1111/j.1471-0528.2008.01703.x 18410658

[9] Colson C Aubry E Cartigny M Remy AA Franquet H Leroy X. SF1 and spleen development: new heterozygous mutation, literature review and consequences for NR5A1-mutated patient's management. Clin Genet. (2017) 92:99–103. doi: 10.1111/cge.12957, PMID: 28032338

[10] Smith OE Roussel V Morin F Ongaro L Zhou X Bertucci MC. Steroidogenic factor 1 regulation of the hypothalamic-pituitary-ovarian Axis of adult female mice. Endocrinology. (2022) 163:bqac028. doi: 10.1210/endocr/bqac028, PMID: 35247045 PMC8974829

[11] Sepponen K Lundin K Yohannes DA Vuoristo S Balboa D Poutanen M. Steroidogenic factor 1 (NR5A1) induces multiple transcriptional changes during differentiation of human gonadal-like cells. Differentiation. (2022) 128:83–100. doi: 10.1016/j.diff.2022.08.001, PMID: 36114074

[12] Luo X Ikeda Y Lala DS Baity LA Meade JC Parker KL. A cell-specific nuclear receptor plays essential roles in adrenal and gonadal development. Endocr Res. (1995) 21:517–24. doi: 10.3109/07435809509030469, PMID: 7588417

[13] Yu BQ Liu ZX Gao YJ Wang X Mao JF Nie M. Prevalence of gene mutations in a Chinese 46,XY disorders of sex development cohort detected by targeted next-generation sequencing. Asian J Androl. (2021) 23:69–73. doi: 10.4103/aja.aja_36_20, PMID: 32985417 PMC7831832

[14] Wang H Zhang L Wang N Zhu H Han B Sun F. Next-generation sequencing reveals genetic landscape in 46, XY disorders of sexual development patients with variable phenotypes. Hum Genet. (2018) 137:265–77. doi: 10.1007/s00439-018-1879-y, PMID: 29582157

[15] Camats N Fernandez-Cancio M Audi L Schaller A Fluck CE. Broad phenotypes in heterozygous NR5A1 46,XY patients with a disorder of sex development: an oligogenic origin? Eur J Hum Genet. (2018) 26:1329–38. doi: 10.1038/s41431-018-0202-7, PMID: 29891883 PMC6117353

[16] Sekido R Lovell-Badge R. Sex determination involves synergistic action of SRY and SF1 on a specific Sox9 enhancer. Nature. (2008) 453:930–4. doi: 10.1038/nature06944, PMID: 18454134

[17] Lin L Philibert P Ferraz-de-Souza B Kelberman D Homfray T Albanese A. Heterozygous missense mutations in steroidogenic factor 1 (SF1/Ad4BP, NR5A1) are associated with 46,XY disorders of sex development with Normal adrenal function. J Clin Endocrinol Metab. (2007) 92:991–9. doi: 10.1210/jc.2006-1672, PMID: 17200175 PMC1872053

[18] Zhang W Mao J Wang X Zhao Z Zhang X Sun B. The genetic spectrum of a Chinese series of patients with 46, XY disorders of the sex development. Andrology. (2024) 12:98–108. doi: 10.1111/andr.13446, PMID: 37147882

[19] Warman DM Costanzo M Marino R Berensztein E Galeano J Ramirez PC. Three new SF-1 (NR5A1) gene mutations in two unrelated families with multiple affected members: within-family variability in 46,XY subjects and low ovarian reserve in fertile 46,XX subjects. Horm Res Paediatr. (2011) 75:70–7. doi: 10.1159/000320029 20861607

[20] Domenice S Machado AZ Ferreira FM Ferraz-de-Souza B Lerario AM Lin L. Wide spectrum of NR5A1‐related phenotypes in 46,XY and 46,XX individuals. Birth Defects Res C Embryo Today. (2016) 108:309–20. doi: 10.1002/bdrc.21145, PMID: 28033660 PMC5347970

[21] Mazen I Abdel-Hamid M Mekkawy M Bignon-Topalovic J Boudjenah R El Gammal M. Identification of NR5A1 mutations and possible Digenic inheritance in 46, XY gonadal dysgenesis. Sex Dev. (2016) 10:147–51. doi: 10.1159/000445983, PMID: 27169744

[22] Robevska G van den Bergen JA Ohnesorg T Eggers S Hanna C Hersmus R. Functional characterization of novel NR5A1 variants reveals multiple complex roles in disorders of sex development. Hum Mutat. (2018) 39:124–39. doi: 10.1002/humu.23354, PMID: 29027299 PMC5765430

[23] Bieth E Hamdi SM Mieusset R. Genetics of the congenital absence of the vas deferens. Hum Genet. (2021) 140:59–76. doi: 10.1007/s00439-020-02122-w, PMID: 32025909 PMC7864840

[24] Liao H Chen Y Li Y Xue S Liu M Lin Z. CFTR is required for the migration of primordial germ cells during zebrafish early embryogenesis. Reproduction. (2018) 156:261–8. doi: 10.1530/REP-17-0681, PMID: 29930176 PMC6106808

[25] Toure A. Importance of SLC26 transmembrane anion exchangers in sperm post-testicular maturation and fertilization potential. Front Cell Dev Biol. (2019) 7:230. doi: 10.3389/fcell.2019.00230, PMID: 31681763 PMC6813192

